# Networks of age‐related pathologies and APOE ε4: their role in hippocampal degeneration

**DOI:** 10.1002/alz70855_103205

**Published:** 2025-12-24

**Authors:** Klara Gawor, Sam Verrept, Geethika Arekatla, David Wouters, Alicja Ronisz, Celeste Laureyssen, Helena Ver Donck, Bas Lahaije, Simona Ospitalieri, Mathieu Vandenbulcke, Markus Otto, Christine A.F. von Arnim, Estifanos Ghebremedhin, Rik Vandenberghe, Alejandro Sifrim, Kristel Sleegers, Dietmar Rudolf Thal, Sandra O. Tomé

**Affiliations:** ^1^ Laboratory of Neuropathology, KU Leuven, Leuven, Belgium; ^2^ Laboratory of Neurobiology, VIB‐KU Leuven, Leuven, Belgium; ^3^ Laboratory of Multi‐omic Integrative Bioinformatics, KU Leuven, Leuven, Belgium; ^4^ Laboratory of Neuropathology, KU Leuven, Leuven, Vlaams Brabant, Belgium; ^5^ Complex Genetics of Alzheimer's Disease group, VIB Center for Molecular Neurology, Antwerp, Belgium; ^6^ Laboratory of Neuropathology, KU Leuven, Leuven, Vlaams‐Brabant, Belgium; ^7^ UZ Leuven, Leuven, Belgium; ^8^ Martin‐Luther‐University Halle‐Wittenberg, Halle, Germany; ^9^ University Medical Center Göttingen, Göttingen, Germany; ^10^ Goethe University Frankfurt, Frankfurt, Germany; ^11^ Laboratory for Cognitive Neurology, KU Leuven, Leuven, Leuven, Belgium

## Abstract

**Background:**

The hippocampus is one of the earliest brain regions affected in dementia‐related neurodegenerative diseases. While Alzheimer's disease (AD) has traditionally been regarded as the primary cause of hippocampal neuronal loss, other pathologies, such as limbic‐predominant age‐related TDP‐43 encephalopathy neuropathologic changes (LATE‐NC), also play a significant role. Age‐related lesions and the APOE ε4 allele, a major genetic risk factor for dementia, exhibit pathogenic synergy, further complicating the clinicopathological landscape.

**Method:**

We analyzed 480 post‐mortem brains (ages 50–99) and used over 30,000 annotated neurons to train an algorithm for quantifying CA1 neuronal density across the cohort. Additional analyses included assessments of brain weight, cognitive status, and lesions such as Amyloid‐β (Aβ), phosphorylated tau, LATE‐NC, α‐synuclein, cerebral amyloid angiopathy (CAA), vascular lesions, Hirano bodies, and APOE genotyping.

**Results:**

Our findings show that ADNC, LATE‐NC, amygdala‐predominant α‐synuclein, small vessel disease, and atherosclerosis contribute to CA1 neuronal degeneration, whereas Hirano bodies appear protective. Structural equation modeling highlighted a network of interconnected pathologies, with tau, LATE‐NC, and α‐synuclein emerging as primary drivers of hippocampal neuronal loss. Aβ deposits, capillary CAA, and LATE‐NC—but not phosphorylated tau—were directly influenced by APOE ε4.

**Conclusion:**

Hippocampal damage in age‐related dementias is multifactorial, extending beyond the contributions of Aβ and tau, with LATE‐NC playing a central role. APOE ε4 indirectly influences hippocampal degeneration by promoting Aβ‐related pathogenesis and triggering interactions among comorbid pathologies that drive neuronal loss.

